# Tinnitus – aktuelle Entwicklungen: Überblick und Zusammenfassung des Wissensstands 2024

**DOI:** 10.1007/s00106-025-01668-3

**Published:** 2025-09-08

**Authors:** Birgit Mazurek, Kurt Steinmetzger, Benjamin Boecking, Gerhard Hesse, Petra Brueggemann

**Affiliations:** 1 Tinnituszentrum, Charité – Universitätsmedizin Berlin, corporate member of Freie Universität Berlin, Humboldt-Universität zu Berlin, and Berlin Institute of Health, Germany Charitéplatz 1, 10117 Berlin, Deutschland; 2 Tinnitus-Klinik am Krankenhaus Bad Arolsen, Große Allee 50, 34454 Bad Arolsen, Deutschland; 3 Universität Witten-Herdecke, Witten, Deutschland

**Keywords:** Tinnitusmodelle, Pathophysiologie, Diagnostik, Multidiszplinäre Therapie, Überblick, Tinnitus models, Pathophysiology, Diagnostics, Multidisciplinary therapy, Review

## Abstract

Chronischer Tinnitus ist ein häufig auftretendes Symptom des auditorischen Systems, dessen Pathophysiologie aufgrund der Multikausalität (ähnlich anderen chronischen Erkrankungen) noch nicht endgültig geklärt wurde. Multidimensionale Verursachung verlangt interdisziplinäre Diagnostik und individuell angepasste Therapiestrategien. Basierend auf einer PubMed-Suche mit dem Begriff „Tinnitus“ für den Zeitraum September 2023 bis September 2024 wurden für dieses Review zur Zusammenfassung aktueller wissenschaftlicher Fortschritte zum Tinnitus 1079 Treffer gefunden, von denen die wichtigsten für diese Zusammenfassung verwendet wurden. Die Tinnitusforschung und -behandlung hat im vergangenen Jahr vorliegendes Wissen in den Bereichen Pathophysiologie, Diagnostik und Therapie vertieft und erweitert. Aufbauend auf neuen Studien wurden zentrale Mechanismen besser verstanden: Tinnitus entsteht oft durch cochleäre Schäden, die zentrale neuronale Veränderungen und maladaptive Plastizität bewirken. Fortschritte bei der Bildgebung und psychoakustischen Tests werden zu einer präziseren Diagnostik beitragen, während in der Behandlung multidisziplinäre Therapieansätze vermehrt in den Fokus rücken müssen.

## Abstract (en)

Chronic tinnitus is a common symptom of the auditory system. Its pathophysiology remains incompletely understood, primarily due to its multifactorial etiology, which resembles that of other chronic conditions. As a result, effective clinical management requires interdisciplinary diagnostics and personalized therapeutic strategies. A search in PubMed using the keyword “tinnitus” covering the period from September 2023 to September 2024 yielded 1079 publications, of which the most relevant were selected and analyzed for this review summarizing current knowledge. Recent advances in tinnitus research have further refined our understanding of its pathophysiology, diagnosis, and treatment. Tinnitus is frequently triggered by cochlear damage, leading to central neuronal alterations and maladaptive plasticity. Progress in neuroimaging and psychoacoustic testing is improving diagnostic precision, while multidisciplinary treatment approaches are gaining clinical relevance and should be increasingly emphasized.

## Theoretische Modelle und Grundlagen des Tinnitus

### Modelle

**Abb. 1:**
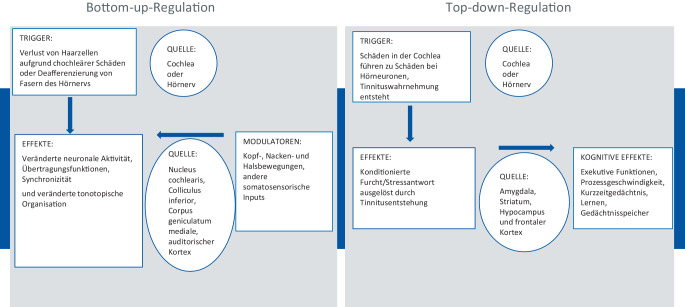
Pathophysiologische Modelle zu Tinnitus. (Mod. nach [1])

Tinnitus, das Phänomen der Wahrnehmung von Phantomgeräuschen ohne äußeren akustischen Stimulus, wird häufig durch cochleäre Schädigungen verursacht, die eine periphere Deafferenzierung zur Folge haben. Diese sensorische Deprivation führt zu plastischen Veränderungen im zentralen Nervensystem, die sich in strukturellen und funktionellen Anomalien wie besonders einer heruntergeregelten Inhibition manifestieren. Von aktuellen Arbeiten zur Pathophysiologie des Tinnitus fasst ein Review die komplexen Prozesse gut zusammen [1]. Es wird ein integratives Modell beschrieben, das die Interaktion von Bottom-up- (sensorische Verarbeitung) und Top-down-Regulation (kognitive Kontrolle) zur Erklärung von Tinnitus heranzieht (Abb. 1). Die Bottom-up-Regulation spiele eine Schlüsselrolle bei der initialen Entstehung des Phantomgeräuschs. Hierbei führen Schäden im peripheren auditorischen System, wie beispielsweise Lärmschäden oder Alterung, zu einer verminderten sensorischen Eingabe. Diese Reduktion der sensorischen Informationen wird von den zentralen auditorischen Prozessen als eine Art „Fehlmeldung“ wahrgenommen, die zu einer Hyperaktivität oder synchronisierten neuronalen Aktivität in den entsprechenden Hirnarealen führt. Die Top-down-Regulation beeinflusst, wie stark das Gehirn diese abnormalen neuronalen Aktivitäten als Tinnitus wahrnimmt und darauf reagiert. Kognitive Prozesse, wie Aufmerksamkeit und emotionale Bewertung, modulieren die Wahrnehmung des Tinnitus. Ein effektiver Top-down-Prozess kann die Wahrnehmung und das Leiden durch Tinnitus mindern, indem er die Aufmerksamkeit von den Phantomgeräuschen ablenkt und die emotionale Belastung reduziert. Therapeutische Ansätze wie kognitive Verhaltenstherapie (KVT) oder Achtsamkeitstraining zielen auf die Modulation der Top-down-Prozesse ab.

Nach dieser Sicht ist Tinnitus als eine Störung sowohl aus Bottom-up- als auch aus Top-down-Prozessen zu verstehen. Während die Bottom-up-Regulation die initiale Entstehung von Tinnitus verursacht, spielt die Top-down-Regulation eine entscheidende Rolle dabei, wie stark das Geräusch wahrgenommen wird und wie sehr es das Leben des Betroffenen beeinträchtigt. Dies ist ein Modell, das den Betroffenen beim Verständnis von leitliniengerechten Therapieangeboten gut helfen kann. Besonders, wenn man deutlich macht, dass nicht Ohrgeräusch selbst, sondern der begleitende Distress den Behandlungsdruck erzeugt [2].

Clarke et al. [3] stellen zwei Modelle gegenüber: Das „Severity of Symptoms“-Konzept (SoS) und das „Correlates of Complaint“-Konzept (CoC). Die SoS-Konzeption des Tinnitus postuliert, dass die Tinnituswahrnehmung Symptome hervorruft, die sich in der Folge auf die die Lebensqualität einer Person auswirken. Das SoS-Konzept hat seine Wurzeln in früheren, rein „peripheren“ Modellen der Tinnitusentstehung, die davon ausgingen, dass die Tinnituswahrnehmung nur infolge der in der auditorischen Peripherie lokalisierten Pathologie entsteht. Tinnitus ist nach diesem „peripheren“ Konzept mit Wahrnehmungsmerkmalen behaftet, die bei PRO (Patient-Reported Outcomes) beobachtbare Symptome verursachen, die sich wiederum auf Funktion und Lebensqualität auswirken. Die SoS-Konzeption sieht den Tinnitus als kausalen Faktor für beobachtete Symptome wie Schlafstörungen, Ängste und Depressionen, Stress, kognitive Schwierigkeiten oder Lautstärke des Tinnituskonstrukts. Im Gegensatz dazu versteht das CoC-Modell diese Beschwerden nicht zwingend als direkte Symptome des Tinnitus, sondern als statistisch assoziierte, jedoch nicht kausal determinierte Phänomene. Es steht im Zusammenhang mit dem theoretischen Konstrukt des *Tinnitusdistress* und reflektiert den Paradigmenwechsel hin zu differenzierten, multikausalen Erklärungsansätzen im aktuellen Tinnitusdiskurs. Mit dem CoC-Konzept entsteht für das Verständnis des Begriffs „Tinnitusschweregrad“ und seiner Beziehung zu weiteren Konstrukten des Tinnitus eine neue Dimension. Um einer theoretischen Unterscheidung und breiteren Darstellung der Tinnitusbelastung Rechnung zu tragen, wird im aktuellen Verständnis die Tinnitusbelastung bzw. der Tinnitusschweregrad als ein fraktioniertes Konstrukt angenommen, das sowohl die Lautheit und Frequenz der Tinnituswahrnehmung als auch die damit verbundene weitere, im Wesentlichen psychologische Belastung umfasst.

Insgesamt ist hierfür das Verständnis von Tinnitus von Bedeutung, dass es sich entsprechend dem CoC-Konzept um eine peripher-zentrale Störung handelt und die über PRO gemessenen Einschränkungen damit *interaktiv* mit dem Tinnitus zusammenhängen. Die „Correlates of Complaint“-Konzeption (CoC) des Tinnitus ist ein zeitgenössisches Erklärungsmodell, das der Beobachtung Rechnung trägt, dass nicht alle Personen mit einem Tinnitusperzept dadurch belastet sind. Sie geht zudem davon aus, dass tinnitusbezogene Beschwerden, wie sie durch patientenberichtete Outcomes (PRO) erfasst werden, nicht notwendigerweise stark mit der psychoakustisch wahrgenommenen Lautstärke des Tinnitus korrelieren. Dies ist bei der Interpretation psychometrischer Ergebnisse als auch von Studien unbedingt zu beachten und muss bei der Festlegung von Behandlungskonzepten berücksichtigt werden.

In einem weiteren Review zur Pathophysiologie des Tinnitus fassen Schilling et al. mehrere neuronale Mechanismen zur Entstehung des Phantomgeräuschs Tinnitus zusammen [4]. Beim Predictive Coding versucht das Gehirn, sensorische Informationen vorherzusagen und Fehler zwischen Erwartung und tatsächlicher Wahrnehmung zu minimieren. Bei Tinnitus könnten fehlerhafter sensorischer Input zu einer Phantomwahrnehmung von Geräuschen führen, obwohl keine äußeren Reize vorhanden sind. Sie nennen dieses Hinzufügen neuronalen Rauschens „stochastische Resonanz“, die zu einer möglichen Verbesserung der Erkennung schwacher Signale führen soll. Die Autoren verwendeten Computermodelle, um zu zeigen, wie diese Prinzipien auf die Entstehung von Tinnitus angewendet werden können, und schlagen als Erklärungsmodell vor, dass beide Mechanismen zusammenwirken, um die auditive Phantomwahrnehmung zu erklären. Die Erklärung bezieht sich dabei auf die Pathophysiologie des Tinnitusperzepts, nicht die begleitende Tinnitusbelastung.

### Neurowissenschaftliche Grundlagenforschung

Außerdem werden funktionelle und strukturelle Hirnveränderungen bei Tinnitus, ein sog. Tinnitusnetzwerk, beschrieben [5]. Husain (2007) beschreibt Tinnitus als Folge funktioneller und struktureller Veränderungen in einem weitreichenden neuronalen Netzwerk, das über die primäre auditorische Verarbeitung hinausgeht. Neben auditorischen Arealen wie dem primären auditorischen Kortex und dem Colliculus inferior sind insbesondere nichtauditorische Strukturen beteiligt, darunter das limbische System (v. a. Amygdala, Hippocampus), der präfrontale Kortex sowie Bereiche des Default Mode Network. Diese Netzwerke modulieren Aufmerksamkeit, Gedächtnis und emotionale Bewertung des Tinnitus und sind mit der Aufrechterhaltung chronischer Tinnituswahrnehmung sowie der subjektiven Belastung verbunden. Husain betont, dass Tinnitus nicht isoliert im auditorischen System entsteht, sondern als dysfunktionales Zusammenspiel mehrerer neuronaler Systeme verstanden werden muss. Die aktuellen Studien liefern hauptsächlich verfeinernde Erkenntnisse und Untersuchungen an verschiedenen Subgruppen.

Anhand von im Ruhezustand erhobenen funktionellen Magnetresonanztomographie(fMRT)-Daten von 100 TinnituspatientInnen und 100 Kontrollpersonen konnte gezeigt werden, dass Patienten mit Tinnitus eine erhöhte Konnektivität im Bereich des Default-Mode-Netzwerks, des Salienznetzwerks und des sensomotorischen Netzwerks aufweisen und diese so für eine Unterscheidung der Probandengruppen genutzt werden kann [6]. Das Default-Mode-Netzwerk **(**DMN) als ein funktionelles Netzwerk im Gehirn, das vor allem dann aktiv ist, wenn eine Person nicht auf externe Aufgaben fokussiert ist – also etwa beim Tagträumen, bei Selbstreflexion, innerem Denken oder dem Abrufen autobiografischer Erinnerungen, zeigt eine erhöhte Aktivität im Ruhezustand in Hirnregionen wie medialer präfrontaler Kortex (mPFC), posteriorer zingulärer Kortex (PCC)/Praecuneus, inferiorer Parietallappen (IPL) und Hippocampus (bei Gedächtnisabruf involviert). Das Salienznetzwerk (SN) ist ein funktionelles Netzwerk im Gehirn, das für die Erkennung und Bewertung bedeutungsvoller Reize – sowohl aus der Umwelt als auch aus dem Körperinneren – verantwortlich ist. Es spielt eine zentrale Rolle bei der Aufmerksamkeitslenkung, indem es zwischen internen (z. B. Gedanken) und externen (z. B. Geräuschen) Reizen vermittelt.

Im Kontext von Tinnitus wird angenommen, dass eine gestörte Interaktion zwischen dem DMN und anderen Netzwerken (wie dem auditorischen oder salienten Netzwerk) zur anhaltenden Wahrnehmung und Bewertung des Tinnitus beiträgt. Beispielsweise könnte eine übermäßige Internalisierung der Tinnituswahrnehmung durch das DMN dessen saliente Bedeutung verstärken und damit das Leiden erhöhen. Das sensomotorische Netzwerk spielt eine zentrale Rolle bei der Erkennung und Verarbeitung von Sinneseindrücken sowie bei der Planung und Ausführung motorischer Funktionen. Bei Tinnitus scheint dieses Netzwerk insbesondere bei der körperlichen Modulation des Tinnitus (z. B. durch Kieferbewegungen, Nackenverspannungen) involviert zu sein. Es umfasst anatomisch den primären somatosensorischen Kortex (S1), den primären motorischen Kortex (M1). das supplementär-motorische Areal (SMA), den posterioren Parietalkortex (v. a. Areale BA 5 und 7), das Zerebellum, die Basalganglien und den Thalamus als sensorischen Relaiskern. Frühere Studien haben gezeigt, dass die funktionellen Verbindungen zwischen dem sensomotorischen Netzwerk und dem Kleinhirnnetzwerk sowie dem exekutiven Kontrollnetzwerk bei Tinnituspatienten abnahmen. Dies führt zur Annahme, dass dies ein möglicher Mechanismus für die Komorbidität zwischen Tinnitus und Hörverlust sein könnte und das Tinnitusperzept damit weniger gut weggefiltert werden kann. In Analogie zu psychiatrischen Störungen besteht das Salienznetzwerk hauptsächlich aus dem anterioren zingulären Kortex. Es wird angenommen, dass abweichende funktionelle Konnektivität zwischen dem Salienznetzwerk und anderen Netzwerken für den Stress von Tinnitusbetroffenen verantwortlich ist. Funktionelle Konnektivität bezeichnet im Kontext des Tinnitusnetzwerks die zeitliche Korrelation der neuronalen Aktivität zwischen verschiedenen Hirnregionen – etwa zwischen auditorischen, limbischen und attentionalen Arealen – ohne direkten anatomischen Zusammenhang. Beim Tinnitus zeigt sich eine veränderte funktionelle Konnektivität, die mit der chronischen Wahrnehmung und der emotionalen Bewertung des Tinnitus in Verbindung steht.

Aktuell konnte anhand struktureller MRT-Daten gezeigt werden, dass anteriore Teile der Insula bei chronischen TinnituspatientInnen ein geringeres Volumen der grauen Substanz aufweisen als bei Patienten mit erst kürzlich aufgetretenem Tinnitus [7]. Allerdings wurde für die untersuchten strukturellen und funktionellen Unterschiede kein signifikanter Zusammenhang mit dem Grad des Tinnitusdistress gefunden. Die Studie zeigt, dass die anteriore Insula eine zentrale Rolle in der emotionalen Verarbeitung und Aufmerksamkeitslenkung bei chronischem Tinnitus spielt. Dabei ist sie besonders in der Umwandlung von akutem zu chronischem Tinnitus involviert, indem sie verstärkt mit anderen Hirnregionen kommuniziert, die für die sensorische und emotionale Verarbeitung zuständig sind.

fMRT-Analysen von TinnituspatientInnen mit (*n* = 26) und ohne (*n* = 34) affektive Störungen zeigen im Vergleich zu einer Kontrollgruppe (*n* = 40) signifikante strukturelle und funktionelle Veränderungen in der Amygdala, besonders in der Subgruppe mit affektiven Störungen [8]. Unterschiede bestanden vor allem hinsichtlich der Intaktheit der weißen Substanz und funktionaler Konnektivität.

Unterschiede kortikaler Aktivierungsmuster zwischen TinnituspatientInnen mit und ohne Hyperakusis wurden von der Tübinger Arbeitsgruppe untersucht [9]. Unter Verwendung verschiedener neurowissenschaftlicher Mess- und Analysemethoden berichten die AutorInnen von unterschiedlichen kortikalen Aktivitätsmustern bei TinnituspatientInnen mit und ohne Hyperakusis. Unter anderem gab es Hinweise auf verminderte Aktivität im auditorischen Kortex in der Gruppe mit Hyperakusis, wenn der Tinnituston präsentiert wurde.

Ein Zusammenhang zwischen Tinnitus und Hörminderung ist gut belegt. Eine britisch-amerikanische Arbeitsgruppe betont nun die Rolle des Hippocampus bei der Persistenz von Tinnitus im Gedächtnis als auditives Objekt [10].

Ein Beispiel für bildgebende Untersuchungen an 64 TinnituspatientInnen mit Normakusis im Vergleich zu 44 normalhörigen Kontrollen kommt von einer chinesischen Arbeitsgruppe [11]. Bei TinnituspatientInnen ohne Hörverlust zeigen sich signifikante Veränderungen in der funktionellen Konnektivität des anterioren Kortex und anderen Gehirnregionen, die mit emotionaler Verarbeitung und Aufmerksamkeit in Zusammenhang stehen. Die molekulare Analyse des anterioren zingulären Kortex zeigte abnormale Expressionsmuster von Genen, die mit neuronaler Erregung, Synapsenbildung und Stressreaktionen assoziiert sind. Diese molekularen Veränderungen könnten zur Pathophysiologie von Tinnitus beitragen, indem sie z. B. eine (genetisch) erhöhte Stressvulnerabilität anzeigen, die das Ohrgeräusch dann u. U. als Belastung erleben lässt und eine neutrale Bewertung des Phantomgeräusches verhindert.

Es wurden mehrere Reviews publiziert, die methodenspezifisch vorhandene Studien reanalysieren. Husein et al. [12] fassen in einem Überblick zusammen, dass Tinnitus nicht nur die sensorische Wahrnehmung betrifft, sondern auch Auswirkungen auf funktionelle Konnektivität von neuronalen Netzwerken hat, die wiederum emotionale und psychologisch-kognitive Prozesse regulieren. Der Schweregrad des Tinnitus spielt eine wesentliche Rolle bei diesen neuronalen Veränderungen.

Die systematische Übersichtsarbeit von Jimoh et al. [13] reviewt 17 Studien zu Gehirnregionen, die mit der Pathologie von Tinnitus und der Kompensation während der Aufgabenbewältigung in Verbindung stehen. Bei Tinnitus zeigen insbesondere die auditorischen Kortexregionen, das limbische System und der präfrontale Kortex abnormale Aktivitätsmuster als auch strukturelle Veränderungen. Während der Ausführung von Aufgaben können bestimmte Gehirnregionen aktiv versuchen, die Auswirkungen des Tinnitus zu kompensieren.

Insgesamt wird mit Methoden der funktionellen Bildgebung zur Abbildung veränderter Hirnkonnektivität bei Tinnitus viel veröffentlicht. Dabei werden in der aktuellen Literatur vor allem Spezifizierungen bekannter Mechanismen und Subgruppenuntersuchungen publiziert, die nur zum Teil direkter klinischer Anwendung zugänglich sind. Vor allem wird nicht eindeutig belegt, ob die gefundenen Veränderungen durch den Tinnitus oder doch durch Hörveränderungen und Hörstress hervorgerufen werden. *Es ist meist auch unklar, wie die beschriebenen Veränderungen mechanistisch zur Tinnitusentstehung beitragen könnten*. Für den Tinnitusdistress scheint vor allem die Konnektivität zwischen präfrontalem Kortex und Amygdala interessant zu sein.

### Tierexperimentelle Studien

Auch tierexperimentell wird mit anderen methodischen Möglichkeiten und unterschiedlichen Tinnitus-Verhaltensmodellen hirnfunktionell und -strukturell weiter an Pathomechanismen zu Tinnitus geforscht. An aktuellen Arbeiten ist eine Studie, veröffentlicht in *Hearing Research* [14], hervorzuheben, wo sich bei Ratten mit verhaltenserfassten Anzeichen von Tinnitus ein signifikanter Anstieg der Aktivität in tieferen Schichten des primären auditorischen Kortex zeigte im Vergleich zu gesunden Kontrolltieren. Dies wird im Einklang mit früheren Studien interpretiert, die eine erhöhte Aktivität des auditorischen Thalamus bei Tinnitus zeigten, da die tieferen kortikalen Schichten thalamischen Input erhalten.

Pak et al. [15] zeigten durch elektrische Stimulation der Oberfläche des auditorischen Kortex bei Mäusen mit lärminduziertem Hörverlust eine kortikale Oberflächenplastizität, insbesondere in den Bereichen des primären und sekundären auditorischen Kortex. Veränderte Neuronenaktivität und synaptische Verbindungen führen zu einer Reorganisation der tonotopen Karte. Diese neuronale Umstrukturierung wird als Mechanismus angesehen, der zur Linderung von Tinnitus beiträgt, da sie eine Anpassung an das veränderte sensorische Umfeld ermöglicht. Die Tiere zeigten nach der Intervention geringere behaviorale Anzeichen für Tinnitus.

Tierstudien bleiben mit ihren besonderen Untersuchungsmethoden wichtiger Teil der Grundlagenforschung zu Tinnitus, auch wenn sie bezüglich der Übertragbarkeit auf Menschen wegen z. B. der Erfassung und Bedeutung von Komorbiditäten sicher begrenzt sind. Beide hier aufgeführten Arbeiten bestätigen bekannte neuroplastische Veränderungen der Tonotopie oder neuronalen Hyperaktivität bei Tinnitus in primären und sekundären akustischen Kortexarealen.

## Epidemiologie

Die weltweite Prävalenz von Tinnitus variiert je nach Studie und Bevölkerungsgruppe, liegt jedoch im Durchschnitt zwischen 10 und 15 % aller Erwachsenen. Die grundlegende Studie von Biswas et al. [16] zeigt: In bestimmten Bevölkerungsgruppen, insbesondere bei älteren Menschen und Personen mit Hörverlust, kann die Prävalenz höher sein, mit Berichten von bis zu 30 % in der Altersgruppe über 65 Jahre. Häufige Risikofaktoren sind Lärmbelastung, Alter und otologische Erkrankungen. Zudem wurde festgestellt, dass Tinnitus häufig mit anderen gesundheitlichen Problemen wie Schlafstörungen, Angst und Depression assoziiert ist. Neue US-amerikanische Zahlen [17] zur Prävalenz (11,2 %) und den Risikofaktoren für Tinnitus (Daten von 2014, *n* = 36.697) zeigen keine Prävalenzunterschiede bezüglich Geschlecht und Ethnizität, wenn Alter, Vorerkrankungen und Lärmexposition berücksichtigt werden.

Aktuelle Kosten der Tinnituserkrankung in unterschiedlichen europäischen Ländern sind von einer internationalen Arbeitsgruppe veröffentlicht [18]: Die Studie von Jarach et al. zeigt, dass Betroffene (*n* = 679) erhebliche Eigenkosten für Diagnostik, Behandlungen und Hilfsmittel wie Hörgeräte tragen. Die Höhe der Ausgaben variiert stark zwischen den europäischen Ländern. Besonders bei nichtmedikamentösen Behandlungen wie kognitiver Verhaltenstherapie, Tinnitus-Retraining-Therapie und Physiotherapie müssen Betroffene häufig eigene finanzielle Mittel aufbringen. Auch die Kosten für Hörgeräte werden nur teilweise von den Krankenkassen übernommen. Diese Studie bietet zum ersten Mal Einblicke in die Zusatzausgaben von Personen mit Tinnitus. Die Out-of-Pocket(OOP)-Kosten wiesen je nach Schweregrad erhebliche Unterschiede auf und beliefen sich in den untersuchten Ländern auf mehr als 17 Mrd. € in einem Jahr. Die OOP betrugen im europaweiten Durchschnitt 368 €, 728 € und 1492 € für leichten, mittelschweren bzw. schweren Tinnitus, mit jährlichen Ausgaben von 565 € für Personen mit verschiedenen Formen des Tinnitus: 209 € für Arztbesuche, 93 € für Behandlungen, 16 € für Medikamente, 64 € für hörunterstützende Systeme und 183 € für Akupunktur, Homöopathie und Osteopathie. In Bezug auf die finanzielle Belastung steht Tinnitus damit in einer Reihe mit den anerkannten führenden Erkrankungen und Beeinträchtigungen. Insgesamt zeigt die Studie, dass die Eigenkostenbelastung für TinnituspatientInnen auch in Deutschland beträchtlich ist, besonders für langanhaltende oder chronische Fälle, da die Gesundheitsversorgung nicht alle Kosten deckt.

Einflüsse von Lärm wurden 2024 über Zusammenhänge von Umweltlärm und dem Auftreten von Tinnitus sowie Tinnitusdistress anhand einer großen Stichprobe (*n* = 6813) genauer untersucht [19]. Die vorherrschenden Quellen der Lärmbelästigung waren neben berufsbedingten Faktoren Umwelteinflüsse wie Flugzeuge, Nachbarschafts- und Straßenverkehrslärm.

Eine australische Studie untersucht die Auswirkungen von Arbeitsplatzlärm und ototoxischen Chemikalien bei 4970 Arbeitern (Survey von 2017–2018) [20]. Etwa 13,5 % der Studienteilnehmer berichteten über Tinnitussymptome, wobei die Prävalenz in bestimmten Bevölkerungsgruppen, wie z. B. älteren Menschen und solchen mit Hörverlust, höher war. Es wurde ein signifikanter Zusammenhang zwischen Tinnitus und Faktoren wie exzessiver Lärmbelastung, Hörschäden und psychischen Gesundheitsproblemen (z. B. Angst und Depression) konstatiert, wobei Lärmbelastung, sowohl beruflich als auch in der Freizeit, als wichtiger Risikofaktor identifiziert wurde. Berufe im Bauwesen, in der Musikindustrie sowie Aktivitäten wie lautes Musikhören und häufige Besuche in lauten Umgebungen erhöhen das Risiko, an Tinnitus zu erkranken. Die Studie hebt die Notwendigkeit hervor, Gehörschutz und andere präventive Maßnahmen zu fördern, um das Risiko von Tinnitus durch Lärmbelastung zu reduzieren.

Auch bei Kindern führen Lärmbelastung und Hörverlust vermehrt zu Tinnitus (allgemeine Prävalenz 10,5 % Tinnitus und 3,2 % Hyperakusis bei 9‑ bis 12-Jährigen), wie in einer belgischen Studie gezeigt wurde [21]. In einer weiteren Studie werden die multidisziplinären Auswirkungen von Tinnitus, aber auch der Misophonie bei Kindern aufgezeigt [22]. Kinder mit Tinnitus und Misophonie zeigen signifikant höhere Raten von psychischen Symptomen wie Angst und Depression im Vergleich zu Kindern ohne diese Störungen.

In einer Kohortenstudie mit Daten aus der UK Biobank wurden an 140.146 Teilnehmern Zusammenhänge zwischen Tinnitus und dem Risiko von kardiovaskulären Ereignissen und der Gesamtmortalität untersucht [23]. Es zeigte sich, dass nach Bereinigung um Störfaktoren das Vorhandensein von Tinnitus mit einer höheren Inzidenzrate für kardiovaskuläre Ereignisse (HR = 1,057, 95 %-KI [Konfidenzintervall]: 1,017–1,099, *p* = 0,005), Myokardinfarkt (HR = 1,139, 95 %-KI: 1,061–1,222, *p* < 0,001) und Gesamtmortalität (HR = 1,053, 95 %-KI: 1,003–1,105, *p* = 0,038) verbunden war. Es wurde jedoch kein signifikanter Zusammenhang zwischen Tinnitus und Schlaganfall oder Sterblichkeit aufgrund von Herz-Kreislauf-Erkrankungen festgestellt. Eine Subgruppenanalyse ergab, dass der Zusammenhang zwischen Tinnitus und den kardiologischen Ereignissen bei Frauen, Teilnehmern mit erhöhtem BMI und solchen ohne Hörprobleme, Depression oder Angstzustände signifikant war. Dabei muss keine kausale Korrelation von Tinnitus und kardiovaskulären Ereignissen vermutet werden, sondern der Zusammenhang erklärt sich indirekt, z. B. über die Stressachse. Chronischer Distress verstärkt sowohl die Tinnituswahrnehmung und kann auch zu kardiovaskulären Veränderungen führen.

Die aktuellen epidemiologischen Studien bestätigen und verfeinern die bekannten Prävalenzen, zeigen aber auch Unterschiede zwischen Ländern und Bevölkerungsgruppen auf. Der Einfluss bekannter Risikofaktoren wie Lärm, kardiovaskulärer oder psychischer Belastung, jetzt auch auf Kinder und Jugendliche, wird anhand von Stichproben mit teilweise hohen Fallzahlen verifiziert. Hervorzuheben ist die europäische Kostenstudie von Jarach et al., die die hohe finanzielle Belastung der Tinnitusbetroffenen (einschließlich Deutschland) genau analysiert.

## Diagnostik

Die 2021 aktualisierten nationalen Leitlinien betonen die multidisziplinäre Diagnostik von Tinnitus [24]. Neben detaillierter Erfassung der Krankengeschichte, einschließlich Dauer, Art und Lautstärke des Tinnitus, möglicher Auslöser (z. B. Lärm, Medikamente, Stress) und begleitender Symptome (z. B. Hörverlust, Schwindel) in der Anamnese gehört dazu die klinische Untersuchung. Über audiometrische Messungen wird das Hörvermögen und der Tinnitus (Frequenz und Lautstärke) erfasst, um Hörverluste zu dokumentieren und die Ausprägung des Tinnitus zu charakterisieren. In der psychometrischen Messung kommen standardisierte Fragebögen wie der Tinnitus-Fragebogen (TF) zur Erfassung der Tinnitusbelastung und der psychischen Belastung (Depression, Angst) zum Einsatz. Wegen der besseren internationalen Vergleichbarkeit sollten wohl die gebräuchlichen Fragebögen wie besonders das Tinnitus Handicap Inventory (THI) oder der Tinnitus Functional Index (TFI) verwendet werden. Bei Verdacht auf zentrale oder vaskuläre Ursachen kann eine Bildgebung (z. B. MRT, MR-Angiographie oder CT) erforderlich sein. Besonders im psychoakustischen (Einflüsse auf Hörvermögen), aber auch psychometrischen Bereich (Fragebogenstandards) gibt es dazu aktuell weiterführende Erkenntnisse.

### Psychoakustik

In einer guten schwedischen Studie konnte an 93 PatientInnen mit Tinnitus über partielle Korrelationen (kontrolliert für Hörfähigkeit in den Standardfrequenzen) ein positiver Zusammenhang zwischen hochfrequentem Hörverlust (10–16 kHz) und der auditorischen Subskala des TFI gezeigt werden, dem sog. auditorisch bedingten Tinnitusdistress, jedoch kein Zusammenhang mit der Tinnituslautheit und anderen Aspekten sowie dem generellen Tinnitusdistress [25].

In einer interessanten Pilotstudie [26] zu sensorischen Prozessen, die zur Tinnitusentstehung führen könnten, nutzt eine internationale Forschergruppe ein Paradigma, das sog. konditionierte Illusionen induziert, indem in einer ersten Trainingsphase visuelle und auditorische Stimuli gemeinsam präsentiert werden, während in der Testphase danach der auditorische oder visuelle Reiz gelegentlich ausbleibt. Es wird angenommen, dass eine höhere Falschalarm-Rate bei den 11 TinnituspatientInnen im Vergleich zu 10 Kontrollprobanden nahelegen würde, dass Tinnitus primär ein zentral-kognitives Problem ist. Die präsentierten Ergebnisse bieten aber keine starke Evidenz für diese Hypothese. In einer parallelen Studie zeigt eine explorative Untersuchung der NeuroMod-Datensätze [27] mittels Graphentheorie Zusammenhänge verschiedener Faktoren bei TinnituspatientInnen. Die Analysen identifizierten enge Zusammenhänge („Teilnetzwerke“) für die Faktoren Hörverlust, Unbehaglichkeitsschwellen und Geräuschempfindlichkeit; sowie Tinnituslautheit und -distress, also eine hinlänglich bekannte Aufteilung.

Eine Schweizer Arbeitsgruppe untersuchte psychoakustische Besonderheiten bei Tinnitus in einem Case-Control-Design mit je 25 Versuchspersonen [28]. Es zeigten sich keine Gruppenunterschiede bei einfachen psychoakustischen Tests (z. B. Gap Detection, Frequenz‑/Amplitudenmodulation), aber schlechtere Performance der Gruppe mit chronischem Tinnitus in komplexen Sprachtests (Störschall und Gating) und zudem schlechtere Werte der Tinnitusgruppe im Stroop-Test, der die Ablenkbarkeit und damit die Funktionalität exekutiver Funktionen misst.

Die aktuellen psychoakustischen Untersuchungen liefern damit Hinweise auf Zusammenhänge von Tinnitus und Hochtonschwerhörigkeit sowie Schwierigkeiten der Höraufmerksamkeit. Tinnitusdistress muss damit unbedingt in verschiedene Faktoren zerlegt werden. Mit dem Hochtonhörvermögen korreliert hauptsächlich auditorischer Tinnitusdistress. Es empfiehlt sich daher bei Tinnituspatienten eine Hörmessung einschließlich der hohen Frequenzen (bis 16 kHZ).

### Psychometrie

Bei der Anwendung von Fragebögen und Verfahren zur Messung der subjektiven Belastungen von Betroffenen durch Tinnitus werden sowohl neue Testverfahren als auch Messkonzepte vorgeschlagen. So bietet eine Studie von Ghodratitoostani et al. [29] ein umfassendes Framework zur ganzheitlichen Bewertung und individuellen Behandlung von Tinnitus, das sowohl diagnostische als auch therapeutische Aspekte integriert und auf eine personalisierte Patientenversorgung abzielt.

Clarke et al. [3] evaluieren die Effektivität und Validität gängiger Instrumente zur Messung von Tinnitusschwere und -belastung. Viele etablierte Instrumente sind zwar hilfreich, können jedoch in ihrer Fähigkeit zur genauen Messung von Tinnitusschwere und -belastung eingeschränkt sein. Das in der Arbeit genauer analysierte THI zeigt, dass es sowohl formative als auch reflektive Elemente aufweist, was seine Interpretation und Anwendung komplex machen kann. Der formative Ansatz betont die Rolle der Items als aktive Faktoren, die das Konstrukt beeinflussen. Formative Items definieren das Konstrukt – sie tragen jeweils eigenständig zur Gesamtausprägung bei, ohne notwendigerweise miteinander zu korrelieren. Beispiel: Items zu Schlafstörungen, Konzentrationsproblemen oder emotionaler Reaktivität formieren gemeinsam das Konzept des *Tinnitus-Handicaps*, obwohl sie unterschiedliche Facetten abbilden. Beim reflektiven Ansatz dagegen werden die Items als Repräsentanten eines bereits bestehenden Konstrukts gesehen – in diesem Fall etwa *Tinnitusdistress*. Die Antworten auf die Items sind durch das zugrunde liegende Konstrukt bestimmt und korrelieren typischerweise stark miteinander. Beispiel: Ein Item wie *„Ich bin wegen meines Tinnitus häufig gestresst“* spiegelt direkt das Ausmaß der empfundenen Belastung wider. Beide Ansätze haben unterschiedliche Implikationen für die Konstruktion und Interpretation von Fragebögen und Tests, wie dem THI.

Aazh et al. [30] stellen einen neuen Fragebogen zur Messung des Selbstvertrauens im Umgang mit Tinnitus vor (4C Tinnitus Management Questionnaire), der in Begleitung einer kognitiven Verhaltenstherapie verwendet werden soll. Der Fokus liegt dabei auf Selbstwirksamkeit, daher findet sich nur eine moderate Korrelation mit dem THI. Dieser ist noch nicht deutschsprachig validiert, somit nur im Forschungskontext anzuwenden.

In einem Übersichtsartikel zum Zusammenhang von gesundheitsbezogener Lebensqualität (HRQoL) und Tinnitusdistress wurden 37 Studien mit einer Gesamtstichprobengröße von 33.900 Teilnehmern einbezogen [31]. Die Ergebnisse sind heterogen und nicht eindeutig. Siebzehn Studien zeigten eine signifikante negative Korrelation zwischen tinnitusbedingtem Stress und HRQoL. Zwei Studien wiesen darauf hin, dass die HRQoL durch den tinnitusbedingten Leidensdruck beeinflusst wird. Achtzehn Studien ergaben, dass Patienten mit Tinnitus im Allgemeinen signifikant schlechtere Werte in HRQoL-Fragebögen erzielten als Personen ohne Tinnitus. Neunzehn Studien zeigten, dass Untergruppen von Patienten mit stärkeren Tinnitusbeschwerden oder spezifischen zusätzlichen Beschwerden in den HRQoL-Fragebögen schlechter abschnitten. Personen ohne Tinnitus weisen im Allgemeinen signifikant höhere Werte in HRQoL-Fragebögen auf als Patienten mit Tinnitus. Eine größere Homogenität bei der Auswahl der HRQoL als auch der Tinnitus-Fragebögen würde einen Vergleich zwischen den Studien ermöglichen, der sowohl auf klinischer als auch auf wirtschaftlicher Ebene wertvolle Informationen für die zukünftige Tinnitusbehandlung liefern würde.

Der Entwicklung und Evaluierung von Fragebögen für Subgruppen widmen sich einige Veröffentlichungen. So wurde ein Tinnitusfragebogen für Kinder vorgestellt [32]. Dieselbe Arbeitsgruppe entwickelte und validierte einen kurzen Hyperakusis-Fragebogen mit 14 Items [33]. Eine exploratorische Faktorenanalyse ergab drei Faktoren: Lautheit (47,3 % der Varianz), Schmerz (13,9 %), Angst (9,3).

In einer sehr relevanten Studie wurden Angaben zu auditorischen Symptomen im Zeitverlauf untersucht [34]. Daten aus zwei Online-Surveys mit der gleichen Kohorte (*n* = 6881) zeigen starke Diskrepanzen zwischen den berichteten Angaben: 30 % der Personen, die im ersten Survey über einen Tinnitus berichtet haben (> 5 min/Tag), und sogar 47 % derer, die vorher über Hörschwierigkeiten berichtet haben, gaben im zweiten Survey an, noch nie jene Beschwerden gehabt zu haben.

Insgesamt impliziert dies für die klinische Praxis und Forschung, dass die Instrumente zur Lebensqualität oder audiologischen/psychologischen Faktoren wie Tinnitusdistress dahingehend einzuordnen sind, ob die Items zum Konstrukt Tinnitus, Hyperakusis usw. oder dessen Belastung aktiv beitragen oder Folgen eines feststehenden Konstrukts widerspiegeln. Psychometrische Daten spiegeln immer einen subjektiven Eindruck im Moment wider. Trotz der Entwicklung und des Vorhandenseins guter Instrumente muss in Studien noch besser auf Homogenität und Festlegung der Outcome-Kriterien geachtet werden. Auch 2024 gilt noch immer, dass in psychometrischen Studien Grundprinzipien eines guten Studiendesigns nur unzureichend beachtet werden, insbesondere die Angabe, welcher Aspekt des Tinnitus oder gesundheitsbezogener Lebensqualität bei therapeutischem Nutzen das Hauptergebnis ist.

### Bildgebung

Ende 2023 wurden vom amerikanischen Institut für Gesundheit neue Empfehlungen für bildgebende Untersuchungen bei Tinnitus herausgegeben [35]. Diese Bildgebungsempfehlung unterstreicht ähnlich den nationalen Leitlinien noch einmal die differenzialdiagnostischen Überlegungen bei pulsatilem Tinnitus und zeigt die Eignung neuer Untersuchungsmethoden (wie Angiographie) für die spezifischen Fälle auf. Dies ist für die klinische Praxis gut umsetzbar.

### Biomarker

Laut nationalen Leitlinien [24] können Biomarker bei der Diagnostik von Tinnitus helfen. Bei Hinweisen auf systemische Erkrankungen (z. B. Stoffwechselerkrankungen, Infektionen) sollte eine spezifische Labordiagnostik durchgeführt werden.

Aktuell fand eine internationale Arbeitsgruppe in einem Case-Control-Design mit insgesamt 1622 Versuchspersonen keine relevanten Biomarker – der Schwerpunkt lag auf entzündungsfördernden Proteinen im Blutplasma, die mit chronischem Tinnitus in Verbindung stehen [36].

Bei einer Untersuchung zu Glutamatwerten bei TinnituspatientInnen mittels Magnetresonanzspektroskopie wurde kein erhöhter Glutamatspiegel im Schläfenlappen von TinnituspatientInnen gefunden [37]. In die Studie wurden 52 Teilnehmer mit ähnlichem Hörprofil und wenig Hörverlust eingeschlossen (*n* = 24 unilateraler Tinnitus, *n* = 28 bilateraler Tinnitus, *n* = 25 Kontrollen), Die Autoren waren davon ausgegangen, dass höhere Werte im Vergleich zu Kontrollpersonen eine erhöhte Erregbarkeit von Neuronen in diesem Teil der Hörbahn widerspiegeln würden.

Insgesamt ist aktuell als noch nicht gesichert anzusehen, ob es tinnitusspezifische Biomarker im Blut, Speichel oder Urin gibt. Hinweise darauf sind häufig schwer zu verifizieren und aktuell noch nicht im klinischen Alltag anwendbar. Trotzdem sind weitere Untersuchungen auf diesem Gebiet wünschenswert, da objektive Maße für die Tinnitusbelastung, auch vor dem Hintergrund eines multidimensionalen Modells des Ohrsymptoms, außerordentlich wertvoll für Diagnostik und Therapie sein werden.

### Genetik

Genetische Untersuchungen weisen auf eine signifikante Rolle genetischer Faktoren bei Tinnitus hin. Ein Review von 2024 fasste 31 Studien mit 383.063 Patienten zusammen und identifizierte genetische Varianten, darunter rs2846071 und rs4149577 (TNFRSF1A), die bei lärminduziertem Tinnitus relevant sind [38]. Sequenzierungsanalysen fanden zudem die Gene ANK2, AKAP9 und TSC 2 bei schwerem Tinnitus [39]. Eine Fallserie von Patienten mit ANK2-Varianten zeigte spezifische Endophänotypen, gekennzeichnet durch Hyperakusis, geräuschartigen Tinnitus, Hochfrequenz-Hörverlust und veränderte AMLR-Amplituden, begleitet von variierenden kognitiven und psychischen Symptomen [39]. Eine 2024 veröffentlichte Studie postulierte genetische Unterschiede zwischen Tinnitus und Hörverlust [40]. GWAS-Meta-Analysen mit 596.905 Probanden identifizierten 39 Tinnitus-Loci, die insbesondere mit neuronalen Synapsen und cochleären Strukturen assoziiert sind. Tinnitus zeigt eine höhere Polygenität und breitere Gewebeexpression im Gehirn als Hörverlust und ist zusätzlich mit psychiatrischen Störungen korreliert. Diese Ergebnisse stützen die Definition von Tinnitus als eigenständige, teilweise genetisch abgrenzbare Störung.

Genetische Untersuchungen zu Tinnitus sind hochaktuell, verweisen mit ihrer Spezifik auf mögliche Unterschiede in verschiedenen Bevölkerungsgruppen, und es darf erwartet werden, dass diese in der Zukunft eine Bereicherung im Verständnis der Entstehung und Auswirkung des Symptoms darstellen.

## Komorbiditäten

### Psychologische Aspekte

Tinnitus ist mit zahlreichen Komorbiditäten assoziiert, insbesondere mit affektiven Störungen, Schlafstörungen, Angststörungen, Anpassungsstörungen und somatoformen Störungen [41].

Studien zeigen, dass Tinnitus in Verbindung mit stressreichen Lebensereignissen steht. Eine Erhebung in chinesischen HNO-Kliniken bestätigte 13 tinnitusbezogene Problembereiche, wobei im Vergleich zu westeuropäischen Vorgängerstudien kulturspezifische Unterschiede auftraten [42]. McKenna und Vogt [43] weisen darauf hin, dass die Rolle kritischer Lebensereignisse bei Tinnitus noch nicht umfassend erforscht ist, aber erste Hinweise auf eine Korrelation bestünden.

Eine spanische Studie mit 710 Personen über 55 Jahren zeigt signifikante Zusammenhänge zwischen Hörminderung, Gleichgewicht und Kognition, wobei eine frühe Prävention von Hörverlust empfohlen wird [44]. Wissenschaftlich belegt ist, dass dekompensierter Tinnitus durch psychologische Begleitsymptome verstärkt, aufrechterhalten oder verursacht wird. Dies betont die Notwendigkeit einer personalisierten und individuellen Diagnostik und Therapie.

### COVID-19

COVID-19-Infektionen können laut mehreren Studien die Wahrnehmung von Tinnitus beeinflussen. Eine Kohortenstudie von Saunders et al. [35] zeigt einen Anstieg der Tinnitusprävalenz während der Pandemie, möglicherweise durch Nocebo-Effekte. Weitere Untersuchungen berichten von persistierenden auditiven und vestibulären Symptomen nach COVID-Infektionen [45, 46].

### Hyperakusis

Der Hyperacusis Impact Questionnaire (HIQ) wurde als valides Instrument zur Beurteilung der Hyperakusis-Intensität entwickelt [47]. Audiologische Unterschiede zwischen TinnituspatientInnen mit und ohne Hyperakusis wurden in einer chinesischen Studie nachgewiesen [48]. Eine US-Studie zur Prävalenz von Misophonie unter Studierenden zeigt Korrelationen mit Hyperakusis, weiblichem Geschlecht und Tinnitus [49].

Die Interaktionen zwischen Tinnitus, Hörminderung und Hyperakusis sind von hoher klinischer Relevanz. Aktuelle Studien liefern wichtige Erkenntnisse zur Pathophysiologie und Diagnostik. Neue Fragebögen sollten vor ihrem klinischen Einsatz deutschsprachig validiert und normiert werden.

### Schlafstörungen

TinnituspatientInnen leiden häufig unter somatisch-depressiven Symptomen wie Schlafstörungen, Unruhe und Konzentrationsproblemen, während klassische affektive Symptome seltener auftreten [50]. Eine chinesische Studie fand keine Unterschiede in den kognitiven Leistungen (MoCA) zwischen TinnituspatientInnen mit und ohne Schlafstörungen, jedoch niedrigere Werte im Vergleich zu Kontrollgruppen, was Tinnitus als unabhängigen Risikofaktor für kognitive Defizite nahelegen könnte [51].

**Abb. 2:**
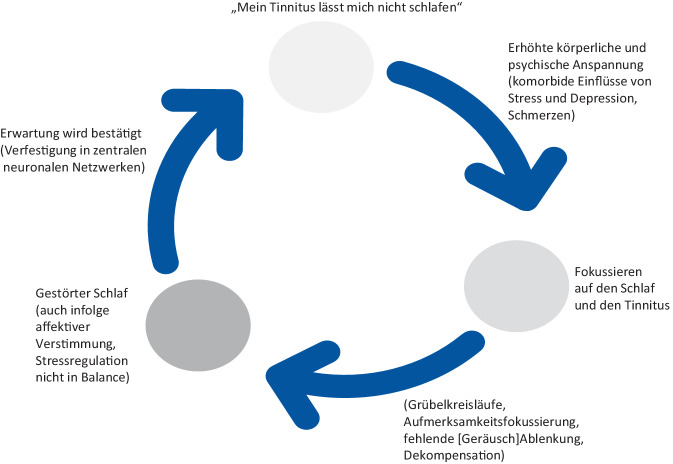
Tinnitus und Insomnie

In großen Kohortenstudien zeigte sich ein signifikanter Zusammenhang zwischen Tinnitus und Schlafproblemen, darunter verkürzte Schlafdauer, Schlaflosigkeit und ungünstige Schlafgewohnheiten [52, 53]. Eine prospektive Analyse ergab ein erhöhtes Risiko für dekompensierten Tinnitus bei Schlaflosigkeit (RR: 2,28, *p* = 0,001) [52]. Diese Daten belegen den klinisch bekannten Zusammenhang zwischen Tinnitus und Schlafstörungen, weshalb eine differenzierte Anamnese der Schlafqualität empfohlen wird (s. auch Abb. 2).

### Kognitive Einbußen und Demenz

Hörverlust zählt zu den 14 modifizierbaren Risikofaktoren für Demenz, deren Prävention die Erkrankungsrate erheblich senken könnte [54]. Eine multizentrische Studie des UNITI-Projekts (*n* = 380) zeigte Zusammenhänge zwischen Tinnitus, Hochtonschwerhörigkeit und kognitiven Einschränkungen [55].

Untersuchungen an 678 Patienten ergaben, dass das Alter nicht nur das Hörvermögen, sondern auch psychologische Faktoren beeinflusst. Während Stress und Angst mit zunehmendem Alter abnahmen, wurde der tinnitusbedingte Leidensdruck durch Hörverlust, Stress und Angst vermittelt [56]. Diese Erkenntnisse unterstreichen die Bedeutung der frühzeitigen Prävention von Hörminderung zur Vermeidung kognitiver Beeinträchtigungen.

## Behandlung

Eine kausale Therapie zur vollständigen Beseitigung von Tinnitus existiert bislang nicht. Tinnitus steht in enger Verbindung zur Hörfähigkeit sowie zu begleitenden Komorbiditäten, die den Tinnitusdistress verstärken [57]. Die S3-Leitlinie empfiehlt für chronischen Tinnitus (≥ 3 Monate) primär Counselling als Basis jeder Therapie [24]. *Counselling umfasst* eine gezielte Informationsvermittlung und häufig interdisziplinäre Beratung *zur Aufklärung der Patientinnen und Patienten über Ursachen, Mechanismen und den Umgang mit Tinnitus.* Nachweislich ist Counselling eine wirksame und evidenzgestützte Basiskomponente der Tinnitusbehandlung, die die edukative und kognitive Auseinandersetzung mit dem Symptom fördert und zur nachhaltigen Entlastung beiträgt. Der Ausgleich eines Hörverlusts durch Hörgeräte oder Cochleaimplantate zeigt eine moderate Evidenz zur Reduktion der Tinnitusbelastung. Die Tinnitus-Retraining-Therapie erfordert eine längere Anwendung; Noiser sind hierfür nicht notwendig. Medikamentöse Behandlungen sowie neuromodulatorische Verfahren, einschließlich repetitiver transkranieller Magnetstimulation, zeigen keine gesicherte Evidenz. Auch Therapien mit Geräuschen oder Musik sind nicht belegt. Die kognitive Verhaltenstherapie (KVT) wird von internationalen Leitlinien als wirksam empfohlen und sollte in spezialisierten Einrichtungen erfolgen, oft multimodal mit hörverbessernden Maßnahmen und Entspannungstechniken kombiniert [24, 57]. Auch eine internetbasierte KVT ist wirksam [84].

Bei strikter wissenschaftlicher Bewertung der Evidenz von Therapieformen bleiben die Empfehlungen flexibel, da besonders auf dem Gebiet der Tinnitustherapie weiter stark publiziert wird [58]. Der Markt für digitale Zusatzanwendungen wächst unübersichtlich [59]. Für spezielle Tinnitusgeräte oder akustische Stimulation existiert bislang kein wissenschaftlicher Wirkungsnachweis [60]. Neue Verfahren bedürfen valider, kontrollierter Studien mit ausreichend großen Stichproben und Placebokontrollen, was bisher selten gegeben ist.

### Akustische Therapie

Die Behandlung eines Hörverlusts ist essenzieller Bestandteil der Tinnitustherapie [57, 60]. Hörgeräte tragen zur Unterdrückung des Tinnitus bei und sollten bereits bei mildem und moderatem Hörverlust eingesetzt werden, insbesondere wegen des Zusammenhangs zwischen Hörvermögen und kognitiven Fähigkeiten [24]. Die Art und Weise der Hörgeräteanpassung beeinflusst die Linderung des Tinnitusdistress maßgeblich. Selbst wenn der Hörverlust gar nicht der Hauptstörfaktor ist, so kann doch ein Ausgleich zu einer deutlichen Verringerung der Tinnituslautstärke und/oder der Tinnitusbelastung führen. Als eine Hauptursache dafür wird neben anderen Ansätzen diskutiert, dass bei Hörverlust die Inhibition für gerade diesen Frequenzbereich zentral heruntergeregelt wird und damit das Ohrgeräusch verstärkt [60]. Eine randomisierte Studie mit drei Behandlungsarmen zeigte, dass nur die Gruppe mit Hörgeräteanpassung nach 12 Monaten eine signifikante Reduktion des Tinnitusdistress verzeichnete [61]. Auch Studien mit Cochleaimplantaten bestätigen eine Verbesserung des Tinnitusdistress bei 60 % der Patienten [62] bzw. zwischen 46 und 95 % bei verschiedenen großen Untersuchungen [63]. Eine schwedische Studie zeigte, dass selbst Patienten mit subklinischer Höreinschränkung von Hörgeräten profitierten [64].

Ein Review von 15 Studien zu auditorischem Training ergab signifikante Vorteile durch Frequenzdiskriminierungs- und Aufmerksamkeitsübungen [65]. Allerdings variierte der Evidenzgrad von begrenzt bis hoch, sodass aus diesen Ergebnissen noch keine Therapieempfehlung resultieren kann. Ein chinesisches Review über Musiktherapie mit 19 Studien bestätigte signifikante Effekte aller untersuchten Musiktherapieformen, wobei die Standard-Musiktherapie als erste Wahl empfohlen wurde [66]. Jedoch wurde die Einbindung in multidimensionale Therapieansätze nicht untersucht.

Neuromodulation soll kortikale Erregungsmuster verändern. Eine kontrollierte Studie zur bimodalen Stimulation (Klangtherapie mit elektrischer Zungenstimulation) zeigte signifikante Effekte nur bei Patienten mit mittelschwerem bis schwerem Tinnitus [67]. Ein Nachweis der klinischen Relevanz fehlt bisher. Ein Review über 24 randomisierte Studien zu Neuromodulation kam zu einer geringen klinischen Wirksamkeit [68]. Die transkranielle Gleichstromstimulation (tDCS) zeigte zwar eine signifikante Verbesserung der Tinnitussymptome im Vergleich zu Kontrollgruppen mit Scheinstimulation, aber die Effektstärke scheint eher gering, und insgesamt wurde in allen eingeschlossenen Studien ein relativ hohes ROB festgestellt [68].

Fazit: Hörgeräte sollten bei Tinnituspatientinnen bereits bei gering- und mittelgradigem Hörverlust in Betracht gezogen werden, da sie nachweislich den Tinnitusdistress reduzieren. Akustische Therapien, insbesondere Diskriminationstraining, erscheinen in Kombination mit Counselling effizient. Neuromodulationsverfahren sind weiterhin in der Entwicklung, ihre klinische Wirksamkeit bleibt jedoch unzureichend belegt [65, 68]. Daher werden sie in der aktuellen Leitlinie nicht empfohlen.

### Psychologische Therapieansätze

2024 wurde die Anwendung kognitiver Verhaltenstherapie (KVT) bei Tinnitus weiter untersucht. Eine Studie mit 696 Patienten zeigte, dass emotionale Bewältigungsstrategien eine wesentliche Rolle bei der Tinnitusbelastung spielen [69]. Patienten mit chronischem Tinnitus neigten zu maladaptiven Vermeidungsstrategien sowie erhöhter emotionaler Verletzlichkeit. Eine Faktorenanalyse derselben Stichprobe identifizierte drei Hauptdimensionen der psychischen Belastung: allgemeine emotionale Belastung, tinnitusassoziierte emotionale Belastung und sozioaudiologische Beeinträchtigung. Die emotionale Belastung war stark mit einem „Verletzlichen Kind“-Modus assoziiert, was auf unerfüllte emotionale Bedürfnisse und Distanzierungsmechanismen als zentrale Behandlungsziele hindeutet [70].

Eine Metaanalyse zu ACT (Acceptance and Commitment Therapy) bei Tinnitus mit drei Studien ergab eine signifikante Reduktion des Tinnitus Handicap Inventory (THI) um 17,67 Punkte im Vergleich zu unbehandelten Kontrollgruppen, was eine klinisch relevante Verbesserung darstellt [71].

Kommentar: KVT bleibt der Goldstandard in der Tinnitustherapie. Ergänzende psychotherapeutische Ansätze, insbesondere aus der Schematherapie oder ACT, zeigen im multidimensionalen Kontext ebenfalls vielversprechende Ergebnisse.

### Medikamentöse und ergänzende Therapien

Die nationalen und internationalen Leitlinien zur Behandlung des chronisch-subjektiven Tinnitus empfehlen keine medikamentöse Therapie. Allerdings können pharmakologische Interventionen wie Antidepressiva und Anxiolytika bei begleitenden Komorbiditäten zur Reduktion der Tinnitusbelastung beitragen [72].

Eine Studie zur Off-Label-Anwendung von Anxiolytika legt nahe, dass kurzfristige Effekte möglich sind, die Anwendung bei chronischem Tinnitus jedoch aufgrund des Nebenwirkungsprofils nicht empfohlen wird [73].

Darüber hinaus wurde in einer Metaanalyse zur Akupunktur mit 36 Studien ein potenzieller Nutzen festgestellt, wenn gleich kontrollierte Studien zur Bestätigung erforderlich sind, da Vorgängerstudien einschließlich Metaanalysen Effekte eher als Placebo einstufen [74]. Eine weitere Untersuchung mit 1593 Jugendlichen ergab eine signifikante Assoziation zwischen Vitamin-D-Spiegel und Tinnitus, jedoch keinen Zusammenhang mit dem Hörvermögen [75]. Eine andere Studie betonte die Bedeutung von Ernährung, Stressbewältigung, körperlicher Aktivität und Schlafhygiene für die Tinnitussymptomatik [76].

Kommentar: Medikamentöse, komplementärmedizinische und ernährungsbezogene Therapieansätze sind nicht Bestandteil der evidenzbasierten Standardtherapie des Tinnitus. Dennoch kann die Berücksichtigung individueller anamnestischer Erfahrungen und Therapieerwartungen bei der patientenzentrierten Behandlung von Nutzen sein. Psychopharmaka sollten gezielt zur Behandlung komorbider affektiver Störungen eingesetzt und durch Fachärzte verordnet werden.

### Multimodale Therapieansätze und Individualisierung

Die aktuelle Forschung unterstreicht die Heterogenität der Tinnitus-Pathophysiologie und die Notwendigkeit individualisierter Therapieansätze. Multimodale Behandlungsstrategien kombinieren HNO-ärztliche, audiologische, psychologische und physiotherapeutische Verfahren. Die zunehmende Digitalisierung eröffnet neue Möglichkeiten, insbesondere durch internetbasierte Therapieformen (IBT). Eine Metaanalyse mit 1574 Patienten zeigte signifikante Verbesserungen der Tinnitusbelastung in der IBT-Gruppe im Vergleich zur Kontrollgruppe [77], Es wurden auch Daten zu Nachteilen von internetbasierter Therapie veröffentlicht, die sich nur als „Behandlungspforte“ und nie ohne begleitende Beratung empfiehlt [59].

Die Individualisierung der Therapie basiert zunehmend auf der Identifikation spezifischer Tinnitusphänotypen. Eine Studie mit 989 Patienten untersuchte vier Hauptgruppen anhand psychologischer und audiologischer Parameter. Während alle Gruppen Verbesserungen nach einer siebentägigen multimodalen Intervention zeigten, profitierten insbesondere Patienten mit hohem psychischem Distress. Gleichzeitig wurden starke Korrelationen zwischen psychologischen Faktoren identifiziert, unabhängig von klassischen audiologischen Merkmalen [78].

Fazit: Die zunehmende Bedeutung personalisierter Therapieansätze zeigt sich in der Differenzierung von Tinnitusphänotypen und der Entwicklung spezifischer Behandlungsstrategien. Eine individualisierte, multidisziplinäre Behandlung unter Berücksichtigung psychosozialer Faktoren stellt eine zentrale Herausforderung der modernen Tinnitusforschung und damit auch -behandlung dar.

## Zukunftsperspektiven

Die Forschung zu Tinnitus fokussiert sich zunehmend auf die neuronalen Mechanismen der multisensorischen Verarbeitung und deren Bedeutung für die Symptomentstehung [79]. Eine differenzierte Betrachtung von Tinnitus als eigenständige Störung könnte neue therapeutische Ansätze eröffnen [80]. Ein zentraler Forschungsansatz ist die Diagnostik von Tinnitusphänotypen zur gezielten Therapieanpassung [81].

Neue Ansätze zur molekularbiologischen Untersuchung des Tinnitus umfassen Gen- und Proteinanalysen in unterschiedlichen Regionen der Hörbahn, wie z. B. im Spiralganglion [82] und Nucleus cochlearis [83]. Eine deutsche Arbeitsgruppe identifizierte durch Anreicherungsanalysen spezifische Proteine, die mit der auditorischen Verarbeitung assoziiert sind, und untersuchte deren potenzielle Rolle bei der Tinnitusentstehung [82]. Die Studie zeigte, dass bei Tinnitus im Nucleus cochlearis vor allem bestimmte Signal- und Transkriptionsproteine (wie BDNF, FOS, JUN und CREB 1) eine zentrale Rolle spielen und dass die biologischen Veränderungen vor allem an den Synapsen und in der Genregulation stattfinden – im Gegensatz zu anderen Hörregionen, wo eher zelluläre Prozesse betroffen sind.

Kommentar: Die Entwicklung effektiver Behandlungsstrategien für Tinnitus bleibt eine zentrale Herausforderung. Psychosomatische Einflüsse spielen eine wesentliche Rolle bei der Krankheitsbelastung, weshalb multimodale und systemmedizinische Ansätze weiter erforscht werden. Die aktuellen Leitlinien betonen die Bedeutung individueller Therapieansätze unter Einbezug evidenzbasierter und multidimensionaler Diagnose- und Behandlungsstrategien.

## Kritische Bewertung und offene Fragen

Zahlreiche Studien bestätigen etablierte Modelle, zeigen jedoch weiterhin Herausforderungen auf:

1. *Uneinheitliche Datenqualität:* Studien zur Epidemiologie variieren in Methodik und Stichproben, was die Vergleichbarkeit erschwert.
2. *Biomarker:* Erste Ansätze zur Identifikation biochemischer Marker sind vielversprechend, aber noch nicht klinisch anwendbar.
3. *Komplexe Wechselwirkungen:* Die Beziehung zwischen Tinnitus und Komorbiditäten wie Schlafstörungen oder kognitiven Defiziten ist nicht vollständig geklärt, was personalisierte Therapien erschwert.
4. *Fehlende Integration:* Erkenntnisse aus Grundlagen- und klinischer Forschung werden nicht immer konsequent verbunden, was den Transfer in die Praxis hemmt.

## Verknüpfendes Fazit

Forschungsarbeiten haben gezeigt, dass Tinnitus ein multidimensionales Phänomen ist, das periphere und zentrale Mechanismen verbindet. Fortschritte bei der Bildgebung, wie fMRT und EEG, ermöglichen Einblicke in neuronale Netzwerke, die emotionale und auditive Verarbeitung verknüpfen. Studien zur psychoakustischen Diagnostik und genetischen Prädisposition liefern ergänzende Perspektiven, um Tinnitus besser zu verstehen. Die Konstrukte „Tinnitus“, „tinnitus disorder“ und „tinnitus distress“ sollten grundsätzlich differenziert werden, um Forschungszielstellungen präziser zu formulieren. Besonders die Kombination von Erkenntnissen aus Neurowissenschaften, Psychologie und Audiologie schafft eine Grundlage für innovative Ansätze.

## Trends und neue Ansätze

Insgesamt ist auch in den nächsten Jahren mit einer Verfeinerung der Ansätze in Diagnostik und Therapie zu rechnen. Eine gründliche HNO-ärztliche Untersuchung und begleitendes Counselling werden aber Grundlage einer leitliniengerechten Versorgung bei Tinnitus bleiben.

1. *Technologie:* Fortschritte in der KI-gestützten Diagnostik, z. B. zur Analyse der Hochfrequenz-Audiometrie, eröffnen neue Möglichkeiten.
2. *Personalisierte Therapie:* Multidisziplinäre Ansätze, die Hörtherapie, kognitive Verhaltenstherapie und bei Indikation Stimulationsoptionen sowie Pharmakotherapie bei Komorbiditäten kombinieren, zeigen wachsende Bedeutung.
3. *Genetik:* Studien zur genetischen Prädisposition betonen die Rolle seltener Varianten und spezifischer Gene in der Pathophysiologie von Tinnitus.
4. *Kosten und Versorgung:* Europäische Analysen heben die finanzielle Belastung hervor und unterstreichen die Notwendigkeit einer verbesserten Gesundheitsversorgung.

## Zukünftige Forschung

Die Forschung sollte folgende Lücken schließen und Schwerpunkte setzen:

- *Robuste Biomarker:* Entwicklung objektiver Parameter für Diagnostik und Therapieerfolg bei Tinnitus.
- *Langzeitstudien:* Untersuchung des natürlichen Verlaufs von Tinnitus und seiner Behandlungsergebnisse.
- *Psychosoziale Dimension:* Bessere Integration psychologischer Faktoren in die Diagnostik und Therapie.
- *Interdisziplinäre Zusammenarbeit:* Förderung von Netzwerken, die Neurowissenschaften, Audiologie und Psychologie vereinen.
- *Prävention:* Verstärkte Forschung zu Risikofaktoren wie Lärmexposition und Präventionsstrategien.

Dieses Update zeigt die Relevanz einer engen Verknüpfung von Grundlagenforschung und klinischer Praxis. Ein integrativer Ansatz ist entscheidend, um TinnituspatientInnen besser zu helfen und innovative Therapien zu entwickeln.

## Abkürzungen

ACT: Acceptance and Commitment Therapy
AMLR: Auditory Middle Latency Response
BA: Brodmann-Areal
BDNF: Brain-Derived Neurotrophic Factor
FOS: Proto-Onkogen c‑Fos (Transkriptionsfaktor)
GWAS: Genome-Wide Association Study
HRQoL: Health-Related Quality of Life
HSIP: High-Score Interaction Protein
MAPK1/3: Mitogen-Activated Protein Kinase 1/3
MRT: Magnetresonanztomographie
NTF3: Neurotrophin 3
NTRK1/3: Neurotrophin-Rezeptoren 1 und 3
PPI: Protein-Protein-Interaktion
STRING: Search Tool for the Retrieval of Interacting Genes/Proteins
Tin: Tinnitus-bezogener Prozess (in bioinformatischer Analyse)
TNFRSF1A: Tumor Necrosis Factor Receptor Superfamily Member 1A
tDCS: Transkranielle Gleichstromstimulation
